# A potential usefulness of ultra-high-resolution computed tomography in quality assurance of remote after-loading system for cervical cancer

**DOI:** 10.1093/jrr/rrae055

**Published:** 2024-08-17

**Authors:** Masashi Kinjyo, Akihiro Nishie, Ryo Kudaka, Shota Nakano, Takuro Ariga

**Affiliations:** Department of Radiology, Graduate School of Medical Science, University of the Ryukyus, 207 Uehara, Nishihara-cho, Nakagami-gun, Okinawa 903-0215, Japan; Department of Radiology, Graduate School of Medical Science, University of the Ryukyus, 207 Uehara, Nishihara-cho, Nakagami-gun, Okinawa 903-0215, Japan; Division of Radiology, University of the Ryukyus Hospital, 207 Uehara, Nishihara-cho, Nakagami-gun, Okinawa 903-0215, Japan; Canon Medical Systems Corporation, 70-1 Yanagi-cho, Saiwai-ku, Kawasaki, Kanagawa 212-0015, Japan; Department of Radiology, Graduate School of Medical Science, University of the Ryukyus, 207 Uehara, Nishihara-cho, Nakagami-gun, Okinawa 903-0215, Japan

**Keywords:** ultra-high-resolution computed tomography, applicator, damage, remote after-loading system, brachytherapy, cervical cancer

## Abstract

Intracavitary brachytherapy with a remote after-loading system (RALS) is performed as a part of radical radiation therapy in cervical cancer. The radiation source is delivered directly through an applicator placed inside the uterus or vagina. Thorough quality control is important to prevent accidents that can lead to serious irradiation error, and an applicator check is one such quality control measure. We experienced a clinical situation in which a small volume of water was observed in the lumen of a post-sterilized applicator on treatment-planning CT. Although the submersion test was negative and no air bubbles emerged from the applicator, ultra-high-resolution computed tomography (U-HRCT) showed a linear crack reaching the inside of the applicator. This abnormality was not identified on treatment-planning CT, which has lower spatial resolution than U-HRCT. In addition, no linear cracks were seen on U-HRCT images of eight other applicators considered to be free from damage. U-HRCT may have superior potential to detect applicator damage and could be useful for quality assurance of the RALS procedure.

## INTRODUCTION

Radical radiation therapy in cervical cancer (CC) uses a combination of external beam irradiation and intracavitary brachytherapy with a remote after-loading system (RALS) [1]. We treat large numbers of patients: last year we conducted over 200 RALS procedures for 75 patients with CC. Unlike external beam irradiation, RALS directly delivers the radiation source through an applicator placed inside the uterus and vagina. Mistakes in the procedure can lead to serious irradiation error; thus, our institution implements thorough quality control according to the guideline proposed by Japanese Society for Radiation Oncology [2]. In addition, we also scan the applicator actually being used in the treatment-planning CT to detect any damage to the instrument. As a result of thorough quality control, about one damaged applicator per year, probably due to age-related deterioration, has been discovered.

Ultra-high-resolution computed tomography (U-HRCT) can provide CT images with improved in-plain spatial resolution and very thin slice thickness reconstruction. Therefore, it is capable of delineating fine structures and detecting abnormalities. U-HRCT has been clinically applied in several different fields including chest, temporal bone and vascular imaging [3–5]. For example, Yoshioka *et al*. [5] mentioned that the artery of Adamkiewicz was better depicted with U-HRCT than by conventional HRCT.

Non-destructive testing (NDT) is an analysis technique, which plays a fundamental role in ensuring the integrity of manufactured parts. The greatest strength of NDT is that it allows for the detection of possible hidden defects without compromising the quality of the inspection target. Therefore, we can use the inspection target repeatedly unless damage is detected during the testing process. CT is one of the imaging techniques used for NDT [6] and has been used in various industries including aerospace, automotive, mechanical engineering, electronics, etc.

Given these backgrounds, we hypothesized that U-HRCT might be able to identify minute damage to applicators, providing an alternative method of quality assurance for the RALS procedure.

## MATERIALS AND METHODS

### Applicators used for RALS

We use two types of applicators: plastic and metal. Metal applicators are highly durable and rarely damaged. On the other hand, as mentioned above, about one plastic applicator per year has been damaged. The plastic applicators used in our institution are CT/MRI applicators (Elekta AB, Stockholm, Sweden). These applicators are washed and sterilized after use for RALS.

### Treatment-planning CT scan

Post-sterilized plastic applicators were scanned by CT to search for the presence of damage as part of quality control at our institution, in addition to visual assessment of the presence of corrosion on the surface. A helical CT scan was performed with a 16-MDCT scanner (Discovery RT, GE Healthcare Japan, Tokyo, Japan). The field-of-view (FOV) was 282 mm. The scanning parameters were 120 kVp, 100-mAs values, 9.37-mm collimation reconstructed to a slice thickness of 1.25 mm, 512 × 512 reconstructed matrix and a pitch of 0.938. Image reconstruction was performed using filtered back projection (FBP). Any applicator suspected of being damaged is routinely investigated using the submersion test described in the guidelines.

### U-HRCT scan

A particular applicator suspected of being damaged was scanned on U-HRCT as well. This applicator was used about 400 times during 12 years by estimation although its durable life was 3 years according to the vendor’s report. This CT scan was performed using a 160 multidetector row system (Aquilion Precision; Canon Medical Systems, Tochigi, Japan). The detector matrix has 1792 channels × 160 rows, and each detector element is 0.25 × 0.25 mm at the isocenter. The beam collimation was 0.25 mm × 160 mm at the isocenter. The focal size was 0.4 × 0.5 mm (super high-resolution mode; SHR mode). The image acquisition in SHR mode was as follows: FOV, 119 mm; slice thickness, 0.5 × 160 row helical scan; tube voltage, 120 kVp; tube current, set by the automatic tube current setting; rotation time 0.5 s, beam pitch 0.806 and 2048 × 2048 reconstructed matrix. Image reconstruction was performed using Advanced intelligent Clear-IQ Engine (AiCE). In addition to axial images, coronal and sagittal images were also reconstructed for assessment. Eight applicators considered to be free from damage were also scanned on U-HRCT in this study. Of these eight applicators, six were also used at the same frequency with the damaged one during the same years, while the remaining two about 100 times during 3 years by estimation.

### Additional experiment for an applicator suspected of being damaged

An applicator was connected to an oxygen inhaler through a tube after removal of the cap. We observed if air bubbles emerged from the scratch after submerging it in the water. We defined as ‘positive’ in case of emerging of air bubbles.

## RESULTS AND DISCUSSION

**Fig. 1:**
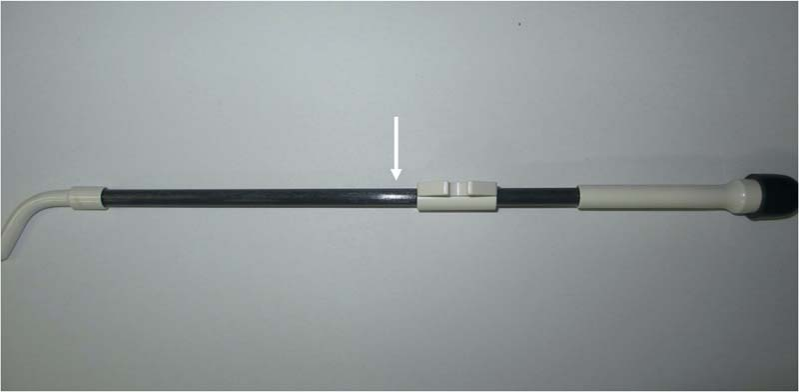
Photograph of a plastic applicator suspected of being damaged. A linear scratch is identified on the surface (arrow).

One day a small water-density volume was observed in the lumen of a plastic post-sterilized applicator on treatment-planning CT (data not preserved). When we visually checked the applicator, we discovered a linear scratch on the surface (Fig. 1). Although the submersion test was performed, no air bubbles emerged. However, we stopped using this applicator due to its possible damage. A few months later, it was scanned by U-HRCT, expecting as a method to confirm minute damage directly.

**Fig. 2:**
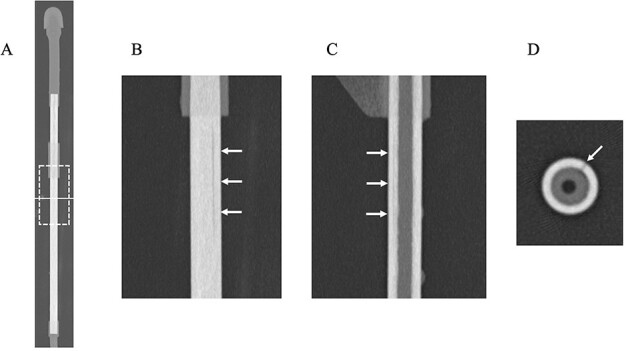
U-HRCT images of a plastic applicator suspected of being damaged (Window Width/Window Level = 6000/900). (**A**) Overall CT image of the applicator. The dotted square shows the scan area of coronal (**B**) and sagittal (**C**) images. The solid line indicates the scan level of axial (**D**) image. (B and C) A linear, hypodense line is identified (arrows). (**D**) The hypodense line reaches deep inside the applicator (arrow).

**Fig. 3:**
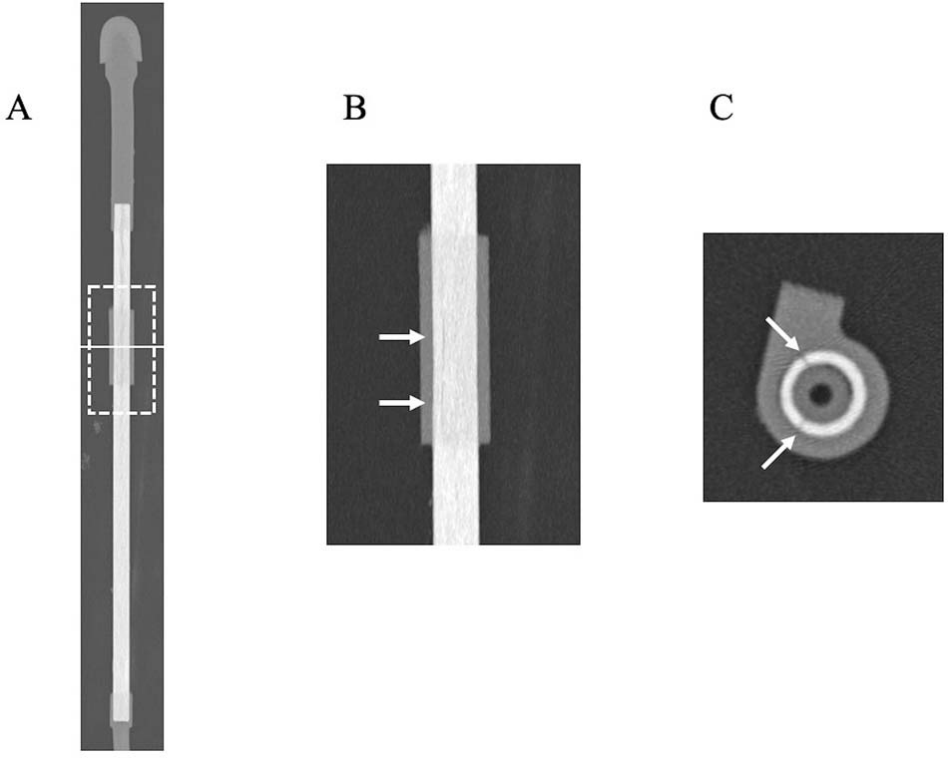
U-HRCT images of the plastic applicator suspected of being damaged at the outer cylinder (Window Width/Window Level = 6000/900). (**A**) Overall CT image of the applicator. The dotted square shows the scan area of coronal (**B**) image. The solid line indicates the scan level of axial (**C**) image. (B) A hypodense line is identified (arrows). (C) Two hypodense lines reach deep inside the applicator at this level (arrows).

**Fig. 4:**
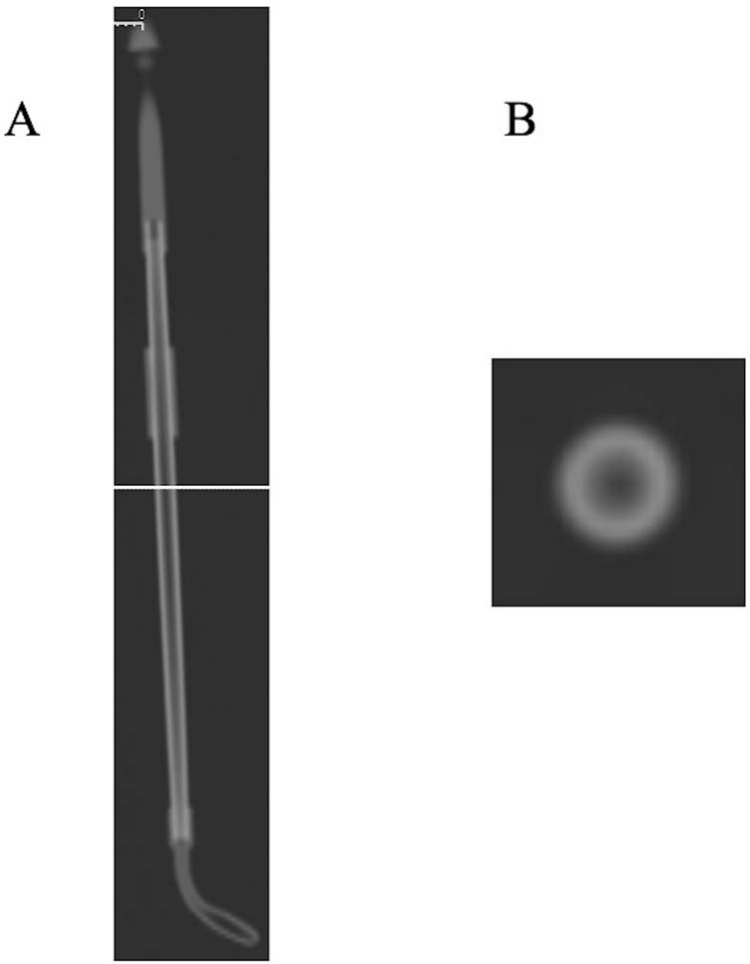
Treatment-planning CT images of a plastic applicator suspected of being damaged at the outer cylinder (Window Width/Window Level = 6000/900). (**A**) The overall CT image of an applicator. The solid line indicates the scan level of the axial (**B**) image, which is equivalent to that of Fig. 2D. (**B**) No hypodense line could be identified. The image is recognized as having lower spatial resolution in comparison with Fig. 2D.

U-HRCT images are shown in Fig. 2. A linear crack was clearly delineated on coronal, sagittal and axial images (Fig. 2B–D). The damage was deep enough to reach the inside of the applicator. U-HRCT images in the region of the outer cylinder are shown in Fig. 3. An axial image clearly showed two linear cracks in the region of the outer cylinder (Fig. 3B). As a result, we judged that the applicator was damaged. The additional experiment performed was also positive. The plausible cause of the damage may be due to exceeded use beyond the durable life. We tried to scan this applicator using treatment-planning CT again. Although the lumen could be identified, overall the images were obscure, and the cracks could not be visualized (Fig. 4). No linear cracks were seen on U-HRCT for the other eight applicators considered to be free from damage.

This study suggested that the damage to a plastic applicator that cannot be detected using the current guideline can be detected using U-HRCT. Although treatment-planning CT missed this minute abnormality, U-HRCT has about one-tenth the pixel size of treatment-planning CT. In addition, the difference in image reconstruction method may contribute to direct visualization of damage. The AiCE adopted in U-HRCT enables noise reduction while preserving spatial resolution with deep learning [7]. Image noise observed in case of FBP was considered to be remarkably reduced. Furthermore, visual assessment and submersion test may not be valid for the region of the outer cylinder because the surface of the applicator is completely covered. However, U-HRCT can also be expected to identify damage to the outer cylinder.

From the above, periodical check-up of applicators used with U-HRCT, which is considered to be equivalent to NDT, could be an advantageous method to maintain the safety of RALS. This case may be an example in which a new imaging technique could contribute to the clinical practice of radiotherapy as well.

This study has a few limitations. First, the reason why the submersion test was negative is unclear. A small volume of water density liquid was confirmed in the lumen of the applicator after washing on treatment-planning CT. Therefore, air bubbles might have been blocked from emerging from the damaged lumen. Second, this study is an investigation using only one case. About one damaged applicator per year has been discovered at our institution. Because it takes much time to enroll many cases, we decided to report using a single case this time. An investigation using more cases of damaged applicators is necessary in the future. Lastly, the influence of radiation exposure to an applicator by CT scan is unclear. However, even if CT scans are repeated on an instrument, its radiation dose would be exponentially smaller than that from the radiation source it delivers.

In conclusion, U-HRCT may have the potential to detect damage to an applicator and could be useful as a quality assurance measure for the RALS procedure.

## Presentation at a Conference

The content of this manuscript was presented at the 36th Annual Meeting of the Japanese Society for Radiation Oncology (JASTRO 2023).
